# The Safety and Efficacy of Phage Therapy: A Systematic Review of Clinical and Safety Trials

**DOI:** 10.3390/antibiotics11101340

**Published:** 2022-09-30

**Authors:** Helen J. Stacey, Steven De Soir, Joshua D. Jones

**Affiliations:** 1 Public Health, Kings Cross Hospital, Clepington Road, Dundee DD3 8EA, UK; 2 Laboratory for Molecular and Cellular Technology, Queen Astrid Military Hospital, Rue Bruyn, 1120 Brussels, Belgium; 3 Cellular & Molecular Pharmacology, Louvain Drug Research Institute, Université Catholique de Louvain (UCLouvain), Avenue E. Mounier 73, 1200 Brussels, Belgium; 4 Infection Medicine, Edinburgh Medical School: Biomedical Sciences, University of Edinburgh, Chancellor’s Building, 49 Little France Crescent, Edinburgh EH16 4SB, UK; 5 Clinical Microbiology, NHS Tayside, Dundee DD2 1SG, UK

**Keywords:** bacteriophage, clinical trial, efficacy, phage therapy, safety, systematic review

## Abstract

Trials of phage therapy have not consistently reported efficacy. This contrasts with promising efficacy rates from a sizeable and compelling body of observational literature. This systematic review explores the reasons why many phage trials have not demonstrated efficacy. Four electronic databases were systematically searched for safety and/or efficacy trials of phage therapy. Sixteen trials of phage therapy were included, in which 378 patients received phage. These were divided into historical (pre-2000; N = 3; *n* = 76) and modern (post-2000; N = 13; *n* = 302) trials. All 13 modern trials concluded that phage therapy was safe. Six of the 13 modern trials were exclusively safety trials. Seven modern trials investigated both safety and efficacy; efficacy was observed in two. Two of three historical trials did not comment on safety, while adverse effects in the third likely reflected the use of phage preparations contaminated with bacterial debris. None of the historical trials contained evidence of efficacy. The evidence from trials is that phage therapy is safe. For efficacy to be observed a therapeutic amount of the right phage(s) must be delivered to the right place to treat infections containing enough susceptible bacterial cells. Trials that have not demonstrated efficacy have not fulfilled one or more elements of this principle.

## 1. Introduction

Bacteriophage (phage) are viruses of bacteria that were discovered independently in 1915 and 1917 [1,2]. Phages are ubiquitous in the environment, with an estimated 10^30^ phages in the oceans alone [3]. Phages are found wherever there are suitable bacterial hosts, including as part of human commensal flora [4]. We therefore exist and have evolved in constant contact with phages. 

The use of carefully selected naturally occurring lytic phage(s) to treat bacterial infections is known as phage therapy. The first recorded instance of phage therapy occurred in Paris in 1919 [5]. The widespread enthusiasm for phage therapy that ensued in the following decades was rapidly curtailed in the geopolitical West by the mass production of antibiotics, which at the time were highly effective, not impeded by resistance, and easier to make, market and use. However, phage therapy continued to be used with success in Russia, Poland and Georgia, although much of the literature generated is inaccessible to Western audiences [6]. The antibiotic resistance crisis has sparked a phage therapy renaissance in the West, where many patients whose clinical needs cannot be met by antibiotics have been successfully treated with phage therapy [7]. Phages can be employed in pre-formulated off-the-shelf cocktails or on a bespoke, personalised, basis. Because of the species-specific, sometimes even strain-specific, manner in which phages infect bacteria it is important to inform clinical use with in vitro efficacy testing of the phage against a patient’s bacteria; this process is sometimes termed phage sensitivity testing and is analogous to modern antibiotic sensitivity testing. 

There is a compelling body of observational evidence supporting the safety and efficacy of phage therapy [7,8,9,10,11,12,13]. Much of this evidence comes from the treatment of patients who had not responded to antibiotics yet were successfully treated with phage. However, despite compelling observational evidence, modern clinical trials, although having consistently demonstrated safety, have thus far inconsistently demonstrated efficacy. This likely reflects challenges in consistently applying the Western clinical trial model to phage therapy, rather than tangible deficiencies in the efficacy of phage therapy. 

This systematic review will collate and critically appraise the results of all available clinical and safety trials of phage therapy. The objective of this review is not to derive summary statistics about the safety and efficacy of phage therapy. Instead, this review will explore the question of why trials may not have yielded efficacy data in line with compelling observational data. This analysis will inform a subsequent discussion of the challenges presented by clinical trials of phage therapy. 

## 2. Methods

### 2.1. Search Strategy

Three electronic databases were searched for articles published up to and including 18 October 2021: EMBASE (1980–2021), Ovid MEDLINE^®^ Epub Ahead of Print, In-Process & Other Non-Indexed Citations, Ovid MEDLINE^®^ Daily, Ovid MEDLINE and Versions^®^ (1946–2021) and Web of Science (1900–2021). The Web of Science Core Collection Citation Indexes searched were: Science Citation Index Expanded (1900–2021), Book Citation Index– Science (2005–2021) and the Emerging Sources Citation Index (2015–2021). The search was performed using the following terms: (phage OR bacteriophage) AND (trial OR volunteer*). In Ovid these terms were followed by the suffix ‘.mp.’ and they were searched as topics in Web of Science. A study protocol was not prepared or published prior to this study.

### 2.2. Study Selection Criteria

The raw search results were deduplicated using Endnote (version X8.0.1). The remaining studies underwent title and abstract screening. Eligible studies were explicitly clinical or safety trials of phage therapy and were published in the English language. Trial protocols and reports of pre-trial pilot studies were excluded. There were no limitations on study date or location. Studies identified as eligible by title and abstract screening were subsequently accessed in full to ensure they fulfilled the inclusion criteria. Studies which could not be accessed in full, including presentation abstracts, were excluded; their authors were not contacted. Title and abstract and full-text screening were performed independently by the authors (SDS or HJS and JDJ), with discrepancies resolved by agreement or, where necessary, a third author (SDS or HJS). This review was conducted in accordance with the PRISMA (Preferred Reporting Items for Systematic Reviews and Meta-Analyses) guidelines [14], and a PRISMA checklist completed (see Supplementary Files S1 and S2).

### 2.3. Data Extraction and Critical Appraisal

The following information was extracted from each eligible study into a spreadsheet: publication year; author(s); study location; study type; number of patients; pathogen(s); condition details; bacterial sensitivity to phage(s); details of the phage(s) used; trial treatment schedule and route(s), assays used to monitor safety and efficacy and details of other ongoing therapies (e.g., antibiotics) where reported; efficacy findings; comments or data regarding safety and adverse effects. All eligible studies were critically assessed using a Joanna Briggs Institute checklist for clinical trials [15]. Data extraction and critical appraisal were performed independently by two authors (HJS, JDJ), with discrepancies resolved by agreement. The influence of publication bias and selective reporting on the cumulative evidence are considered in the discussion.

## 3. Results

### 3.1. Study Selection and Characteristics

**Figure 1:**
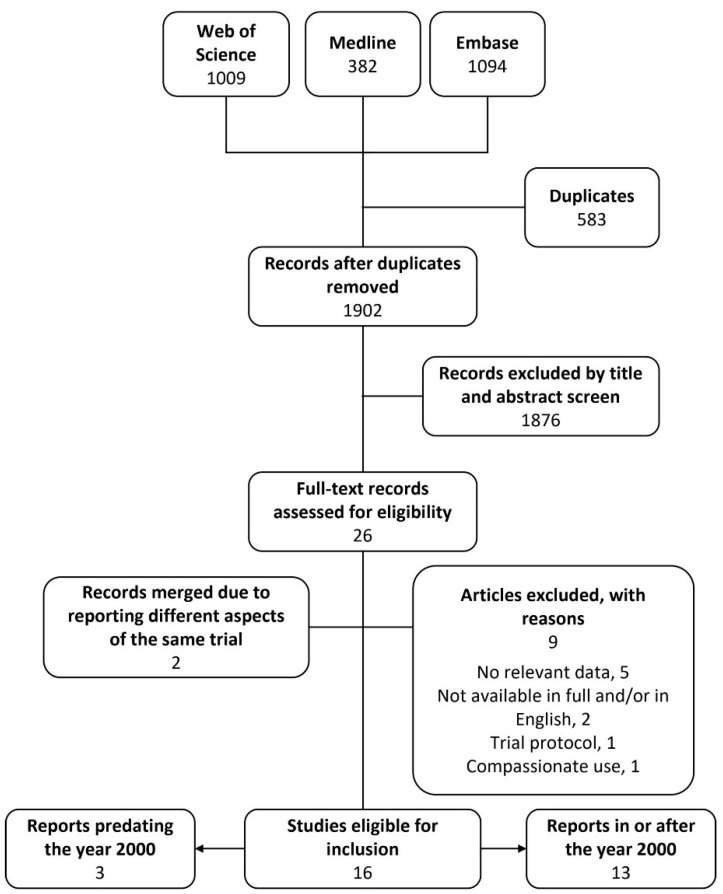
Flow diagram of study selection.

Systematic searching of three databases yielded 2485 articles, from which deduplication removed 583. The titles and abstracts of the remaining 1902 articles were screened against the selection criteria. Title and abstract screening identified 26 eligible articles, nine of which were excluded after full-text screening. Articles were excluded at the full-text stage because they were: not a trial of the safety or efficacy of phage therapy (*n* = 5), not published in English and/or not available in full (*n* = 2), a trial protocol (*n* = 1) or deemed to be compassionate use and not a clinical trial (*n* = 1). Patients in the latter report were treated with intravenous phage for severe staphylococcal infection. Nine patients were treated under an Australian ‘compassionate-use special access scheme’ [16]. Four additional patients were treated under a ‘clinical trial notification scheme’. There was no difference between the way phage was used in patients who received phage under either scheme and we deemed that the ‘trial’ use of phage was therefore more closely aligned with compassionate use than a traditional clinical trial. This study was therefore excluded from analysis in this review. The study selection process is shown in Figure 1. A total of 17 reports of clinical or safety trials were eligible for inclusion, two of which reported different aspects of the same trial and were therefore considered as one trial [17,18]. A total of 16 clinical or safety trials were considered by this review, these were divided into two groups for analysis: historical reports and modern reports from the year 2000 onwards. The data extracted from the trials is shown in Supplementary File S3. While critical appraisal of eligible studies highlighted shortcomings in reporting it did not reveal further grounds to exclude any studies (see Supplementary File S4).

### 3.2. Modern Trials

The 13 modern clinical or safety trials identified were published between 2005 and 2021 [17,18,19,20,21,22,23,24,25,26,27,28,29,30]. The trials were undertaken in Bangladesh and/or Switzerland (*n* = 6), the United States (*n* = 2), France and/or Belgium (*n* = 2), Australia (*n* = 1), Georgia (*n* = 1) and the UK (*n* = 1). Of the 13 trials, four were designated as ‘phase I’, two as ‘phase I/II’ and seven did not contain any explicit designation. The trials covered the administration of phage orally (*n* = 6), topically (*n* = 3), both orally and intra-nasally (*n* = 1), intra-aurally (*n* = 1), intra-nasally (*n* = 1) and by intra-vesicular washing (*n* = 1). No pre-phage gastric neutralisation was employed in any of the seven studies which administered oral phage. However, phage was suspended in weakly alkaline mineral water in five of the seven trials which used orally administered phage. The diverse routes of administration reflect the diverse range of pathogens and clinical conditions to which phages have been applied. Phages were applied as cocktails (*n* = 11), single-phage preparations (*n* = 1) or both cocktails and single-phage preparations (*n* = 1). Six of the 13 trials were safety trials among healthy adults and children. The remaining seven studies examined the efficacy of phage therapy for burn wound infections caused by *P. aeruginosa* and/or *S. aureus* (*n* = 2); diarrhoea caused by *E. coli* (*n* = 1); chronic otitis caused by *P. aeruginosa* (*n* = 1); staphylococcal chronic rhinosinusitis (*n* = 1); complex urinary tract infection caused by various pathogens (*n* = 1); or chronic venous leg ulcer patients in whom the presence of infection was not a criterion for inclusion (*n* = 1). Patients received, or were able to receive, simultaneous antibiotic therapy in three of the seven efficacy trials. Among the seven efficacy trials, prospective investigation of the bacterial sensitivity to phage therapy was undertaken in three trials, retrospective investigation in two and no investigations in two trials. Together, excluding control groups, the modern trials represented the application of phages to 302 patients from the ages of six months old, of which 156 were healthy/uninfected and 146 had a relevant bacterial infection.

### 3.3. The Safety of Modern Phage Therapy

Given the diverse array of phages, treatment regimens and routes of administration, the 13 modern clinical and safety trials included in this review provide a broad overview of the safety of phage therapy. Representing the administration of a range of phages in high concentrations by a variety of routes and, having used a comprehensive array of appropriate safety monitoring assays, all 13 of the modern trials concluded that phage therapy was safe and without phage-related adverse effects. This will be reassuring to readers less familiar with phage therapy. However, that trials have consistently demonstrated the safety of phage administration by a variety of routes is perhaps unsurprising when the evolutionary context is considered. Phages are ubiquitous in the environment and form a significant part of commensal human flora. For example, there are an estimated > 10^9^ phages per gram of human faeces [31]. Phage genomes have been detected in the cerebrospinal fluid of healthy volunteers not treated with phage therapy and in a wide variety of other body fluids [32,33]. Together, this suggests that because humans have co-evolved with high numbers of phages over millennia, naturally occurring phages (whether environmental or therapeutic) are unlikely to elicit adverse immunological effects.

### 3.4. The Efficacy of Modern Phage Therapy

Six of the 13 modern trials included in this review were safety trials and did not evaluate efficacy. Of the remaining seven trials, two demonstrated the efficacy of phage therapy in the context of chronic antibiotic-refractory infections [19,29]. In 2009, Wright and colleagues treated patients with chronic otitis caused by *Pseudomonas* with one dose of a six-phage cocktail and observed significant clinical improvements from baseline and lower bacterial counts in the phage-treated group compared to the control group [19]. More recently, Ooi and colleagues treated patients with chronic rhinosinusitis with a three-phage cocktail for 7 or 14 days and noted that all patients had a reduction in the growth of *S. aureus* and in 2/9 bacterial eradication was achieved [29]. 

The remaining five clinical trials were published over the last seven years and did not adequately demonstrate the efficacy of phage therapy. Examination of these trials shows that in hindsight these results are not a comment on the mechanistic efficacy of phages in killing bacteria, but instead reflect ongoing clinical and technical challenges facing phage therapy trials. 

In 2009, Rhoads and colleagues undertook a phase I safety trial of topical phage therapy among patients with chronic venous leg ulcers [21]. The report stated that the primary goal of the study was to evaluate the safety of the phage cocktail and that the trial was ‘not designed as a formal efficacy study’. Crucially, the presence of infection was not one of the inclusion criteria, meaning it was not clear how many patients actually had an infection and that some patients without an infection received phage. Given this, it is not possible to draw any conclusions about the efficacy of phage therapy from this trial and it is unsurprising that there was no significant difference in the rate or frequency of wound healing between phage-treated and control groups.

In 2014, Rose and colleagues undertook a small-scale clinical trial of topical phage therapy for burn-wound infection [24]. A single dose of 0.03 mL/cm^2^ of phage cocktail was administered to half of a patient’s wound area. However, the patients experienced a delay of up to seven days in admission to the trial. In that time all the patients were receiving appropriate antibiotic therapy. Consequently, comparison of the bacterial loads between the phage treated and standard care halves of the wounds did not reveal any notable difference. Moreover, the authors noted that it was not expected that a single dose of topical phage therapy would elicit any conclusive proof of phage efficacy and rather the significance of the trial lay in its very undertaking. 

In 2016, Sarker and colleagues treated 78 children with diarrhoea, aged 6–24 months, with one of two oral phage cocktails [25]. The treatment was empirical and no prospective analysis of bacterial sensitivity to the phages was performed. Nor had the phage cocktails been tailored to local circulating strains of *E. coli*. No intestinal amplification of phages was observed, including in patients with phage-sensitive strains of *E. coli*, and the diarrhoea was attributed to streptococcal overgrowth in some patients. The authors considered that for those patients with sensitive *E. coli* there were likely too few *E. coli* organisms to sustain sufficient phage replication. Moreover, it is considered to be advantageous to administer gastric neutralisation prior to oral phage therapy [34]. However, gastric neutralisation was not performed in this trial; although the authors did not consider this a limiting factor, stating that children have a higher gastric pH than adults. 

In 2019, the much-anticipated results of the PhagoBurn trial were published, however the trial did not yield evidence of efficacy for several reasons [28]. Crucially, during storage the titre of the phage had dropped from a therapeutic 10^6^ PFU/mL to a subtherapeutic 10^2^ PFU/mL. This rendered PhagoBurn at best a safety trial as, although many phage particles were inactive, the equivalent of 10^6^ PFU/mL of phage proteins were nonetheless administered topically. Aside from the phage concentration, it is notable that the infecting *P. aeruginosa* strains were of varying antibiotic sensitivity which could have complicated delineation of the relative antimicrobial effects of phage and antibiotics. Moreover, during the trial, it was found that three of the 10 participants in the phage group had *P. aeruginosa* colonies resistant to phage on day 0 of the trial. PhagoBurn therefore also illustrates the microbiological complexities associated with phage therapy and varying levels of resistance to antibiotics and phages that have the potential to complicate clinical trial results. 

Most recently, in 2021, Leitner and colleagues published a clinical trial of intra-vesicular phage therapy for patients with complex urinary tract infection [30]. Despite appropriate phage sensitivity analysis, phage therapy was not superior to the placebo, but nor was phage inferior to appropriate antibiotic therapy. The poor efficacy of antibiotic therapy in this trial was surprising, especially given antibiotic sensitivity testing was undertaken. However, the often recurrent and recalcitrant nature of urinary tract infections may reflect the existence of quiescent intracellular reservoirs of uropathogenic bacteria within epithelial tissue that are consequently less accessible to antimicrobial therapeutics [35]. In terms of phage therapy, the authors posited that the absence of efficacy may have reflected the use of a diverse pre-formulated phage cocktail which in vivo translated into low titres of multiple different types of phages, not all of which would have had activity against the causative pathogen. Delivery of an insufficient concentration of phages to the site of infection and potentially low numbers of (potentially inaccessible) bacteria in the bladder epithelium represent significant challenges to intra-vesicular phage therapy for urinary tract infections.

In summary, the four recent clinical trials that have failed to demonstrate the efficacy of phage therapy have done so because they have not achieved the ‘Goldilocks’ constellation of getting the right amount of the right phage(s) to the right place to treat infections containing enough susceptible bacterial cells. If just one of these factors is sub-optimal the efficacy of phage therapy will be dramatically reduced. However, the success of other clinical trials shows that it is possible to achieve a favourable constellation of these factors. However, such can be the variation between infections that achieving the appropriate constellation of these factors is arguably simpler on a case-by-case basis. This explains the notably greater success achieved by personalised clinical applications of phage therapy, in which both the phage(s) and clinical approach are tailored precisely to a patient’s individual clinical needs.

### 3.5. Historical Trials

Three historical trials of phage were identified by the systematic search [36,37,38]. These took place in the US in 1965 and 1966 and in Pakistan, with support from the USSR, in 1971. The two trials in the United States both reported the use of raw staphylococcal phage lysate to treat either recurrent furunculosis or infective childhood asthma. Phage was injected intramuscularly in the 1965 trial and ‘injected’ in the 1966 trial. The later trial in Pakistan reported the use of an oral phage cocktail, supplemented in some cases by intramuscular injection of phage, to treat acute cholera. Neither of the trials in the US reported phage sensitivity testing and although phage sensitivity testing was done in the trial performed in Pakistan it was unclear whether it was pro- or retrospective. Regarding antibiotics, one phage- and one placebo-treated patient received antibiotics in the 1965 trial, all patients in the 1966 trial of phage for infective childhood asthma were able to receive antibiotics if appropriate and the 1971 trial did not report whether patients received antibiotics during the trial. Excluding control groups, the historical trials represented the application of phages to 76 patients, of which all were considered to have a bacterial infection. 

### 3.6. The Safety of Historical Trials

Two of the three historical trials, published in 1966 and 1971, did not comment on safety or adverse effects [36,37]. The US trial published in 1965 reported local reactions (erythema, pain and swelling) to intramuscular injection of raw staphylococcal phage lysate [38]. The two historical trials from the US reported the use of raw staphylococcal phage lysate. The trial from Pakistan reported inactivating any remaining bacteria in the raw lysate and filtering it through asbestos before dispensing into vials.

Historical users of phage were unable to purify their phage preparations. Impure phage preparations, such as raw staphylococcal phage lysate, contain a mixture of dead (lysed) bacterial cells, phage particles and bacterial growth media. Although a reaction to phage cannot be ruled out it is unlikely given the absence of adverse effects from modern clinical trials. Instead, the reactions reported most likely reflect a combination of an injection-site reaction and immune response to the substantial quantity of staphylococcal debris. Similar reactions have been observed in other 20th Century clinical reports of the use of subcutaneously injected raw phage lysates [39,40]. There have not been reports of any such reactions among modern applications of purified anti-staphylococcal phage [1,2,3,4,5,6,7,8,9,10,11,12,13,14,15,16,17,18,19,20,21,22,23,24,25,26,27,28,29,30,31,32,33,34,35,36,37,38,39,40,41,42,43,44,45]. 

### 3.7. The Efficacy of Historical Trials

None of the three historical trials reported evidence of efficacy. Bryant and colleagues evaluated the efficacy of intramuscular phage injections for the treatment of chronic furunculosis among children [38]. It is not surprising that this trial did not demonstrate efficacy. First, this trial did not evaluate the sensitivity of the staphylococcal strains to the phage used. This means that many of the patients may have been treated with an ineffective phage. Second, it is unclear whether the role of alternative pathogens was evaluated and how many of the furuncles may have been caused by bacteria other than *S. aureus*. There is also insufficient information in the report to confirm the nature of the pathologies in question; for example, we now consider acne to be a multifactorial complex pathology, rather than a straightforward bacterial infection [46]. Third, this report evaluated intramuscular (IM) phage injections as a treatment for superficial furunculosis. For phage therapy to be successful, the phages must encounter the bacteria, ideally by being administered to the site of infection. In this case, it is doubtful whether any of the phages administered by IM injection would have reached the target furuncles, with many phages likely being destroyed by the innate immune system at the site of injection or in the draining lymphatic system. 

Wittig and colleagues also evaluated the efficacy of injected staphylococcal phage lysate, in this case for the treatment of infective childhood asthma [36]. The outcome of this trial is similarly unsurprising for several reasons. First, it is unclear which, or whether, symptomatic episodes were caused by staphylococcal infection as no microbiological analyses were reported. Even if we assume staphylococci were the causative agent for some patients, no phage sensitivity analyses were reported. Second, efficacy was measured among the children by parental completion of symptom report cards in which episodes of coughing, fever, wheezing and colds were documented. The trial was conducted during the winter of 1963–64 and multiple other infectious agents could have been responsible for such symptomatic episodes, rendering the measure of efficacy uninterpretable. Third, the finding that there was no difference in the presence of nasopharyngeal *S. aureus* between the two groups reflects the lack of impact that phage administered by IM injection could reasonably be expected to have on nasopharyngeal colonisation, without considering the prior absence of bacterial culture and phage sensitivity analysis. 

In 1971 Marcuk and colleagues evaluated the efficacy of oral phage therapy for the treatment of acute cholera [37]. This trial found that oral phage did not offer significant improvement over standard oral rehydration therapy, while oral tetracycline was superior to phage. The patients were infected with either classical or El Tor strains of *V. cholerae*. Although phage sensitivity testing was undertaken it was not clear whether it was pro- or retrospective. Nonetheless, the results of the phage sensitivity testing showed that while all classical strain isolates were susceptible to the phages used, the El Tor isolates exhibited varying degrees of substantial resistance. The authors noted that it is difficult to predict the phage sensitivity of *Vibrio* strains and suggested incompatibility between the phage and bacterial strain as an explanation for the El Tor results. Of greater importance however, and neatly accounting for the failure of phage therapy among the patients with classical *Vibrio*, was the authors observation of the temporal incompatibility of phages with acute cholera. The authors noted that only small numbers of phages were used relative to the high number of *Vibrio* in the intestine. The authors further noted that the transit time in an ‘actively purging’ cholera patient is sometimes as short as 20 min. Meanwhile, at least 30 min is required to complete a phage replication cycle. Effective phage-mediated elimination of *Vibrio* would therefore have required many rounds of phage replication over several hours, time not afforded by the action of cholera toxin. It’s therefore likely that many phages were simply excreted before they were able to undergo replication. Moreover, it was not reported whether pre-phage gastric neutralisation was performed and it is therefore unclear the extent to which gastric pH may have reduced the amount of phage progressing beyond the stomach.

## 4. Discussion

Together the trials of phage therapy identified in this review provide reassuring data about the safety of phage therapy, affirming the findings of other recent reviews [12,13]. However, at face value, these trials provide a mixed picture about the efficacy of phage therapy. Closer interrogation reveals that limited efficacy arises from clinical and microbiological challenges unique to phage therapy. Table 1 shows that the trials which have successfully demonstrated efficacy have met these challenges.

Notably, despite over 100 years of clinical use, this review identified only 16 clinical or safety trials. This partly reflects the limitations of a systematic review approach: to be eligible for inclusion in this review, trials must have been indexed in full-text form in one of three online databases and published in English. This method, although thorough and appropriate for a systematic review, does not adequately account for the history of phage therapy. Many historical trials will not be indexed in online databases. Moreover, Russian and Georgian clinical trial data is generally not available to Western audiences. This creates a bias in the impression created by examination solely of reports published in Western English-speaking contexts.

The evidence presented by the trials included in this review must also be viewed in the wider context of available clinical evidence, most notably observational clinical data [7,12]. Most of the evidence in favour of the safety and efficacy of phage therapy is derived from case series and case reports; the results of >2200 patients treated with phage therapy outside of a clinical trial setting have been published in English language journal articles since the year 2000 [12]. However, given the long history of clinical phage therapy this figure is almost certainly a gross underestimate of the actual number of patients treated with phage, with some results published prior to the year 2000 and others not being published in English or at all, especially in contexts where phage therapy is already widely accepted. Nonetheless, this significant body of evidence is sometimes met with scepticism because of its observational nature. Although a body of observational evidence may be more likely to contain reports of positive outcomes and less likely to contain negative outcomes (publication biases inherent in such evidence), that such compelling observations have consistently been reported about the safety and efficacy of phage from multiple different sources, and especially in infections refractory to antibiotics, lends substantial weight to these observational findings. 

Nevertheless, the available clinical results paint a reassuring picture of the safety of phage therapy; in keeping with human co-evolution with phages. To the best of our knowledge there have only been 11 instances of possible clinical adverse effects to modern phage therapy, none of which were believed by the report authors to be directly attributable to phage [47,48,49,50,51,52,53,54]. Reassuringly there have also been multiple efficacious and side effect free reports of phage therapy by invasive routes of administration (e.g., intravenous, intra-articular) and in immunocompromised patients [16,45,55,56,57,58]. 

In terms of efficacy, most patients that receive phage therapy do so because they have failed antibiotic therapy. Although it is usual for such patients to continue to receive antibiotics during phage therapy, resolution of infection in the context of antibiotic resistance or tolerance in a manner that coincides with phage therapy is both compelling and consistently observed [7]. Moreover, in most such cases there is also compelling in vitro evidence of efficacy in the form of bacterial phage sensitivity testing. Given the convincing clinical evidence in favour of efficacy it is therefore reasonable to conclude that the failure of clinical trials thus far to demonstrate compelling efficacy is a consequence of a complex inter-play of factors, described above.

Clinical trial evidence has become a central tenet of Western medicine, providing broad reassurance. However, it is important to acknowledge that clinical trial data is not the only form of evidence. As we have observed among the trials discussed above, phage therapy, by its dynamic and biological nature, presents inherent difficulties to the clinical trial model. First, phage preparations will require ongoing adjustment and reformulation to adapt to changing bacterial populations and resistance patterns. For example, ongoing reformulation is performed by the Eliava Institute for their pre-formulated phage cocktails. A clinical trial of a single phage is therefore likely to be uninformative, as, notwithstanding some exceptions, it will likely need to be used in combination with other phages to mitigate bacterial resistance. Likewise, a cocktail of phages subjected to a clinical trial will likely require ongoing reformulation, fundamentally altering the ‘active ingredients’. Notably, personalised phage cocktails present similarly unique challenges to the existing clinical trial model. Second, there are potentially thousands of clinically useful phages in the environment. It would be impossible to put every phage, or combination of phages, through clinical trials for just one type of infection, let alone the breadth of bacterial infections that phage could be applied to. Third, as we have seen with the trials included in this review, successful phage therapy involves the ‘Goldilocks’ constellation of getting the right amount of the right phage(s) to the right place to treat infections containing enough susceptible bacterial cells. This will be easier for some clinical indications than others, which may require a more tailored clinical approach. Consequently, demonstrating efficacy through one-size fits all clinical trial protocols may be more difficult for certain infections. Fourth, as is also evident in this review, there is much variation between existing treatment protocols. This reflects both variation in clinical phage practice and in some circumstances the tailoring of clinical phage protocols to individual patients. For example, joint infections have been successfully treated by daily administration of phage via wound catheter, a single intra-operative administration of phage or simultaneous courses of intra-articular and intravenous phage [44,45,50]. This presents a further challenge to prospective clinical trials. Broad clinical protocols suited to clinical trial methodology may not be the most clinically appropriate approach for individual patients. For trial designers the question is which of the varied treatment protocols to trial. Although trial results may play a part in shaping future clinical practice, it is important that flexibility to deliver more personalised treatment approaches is retained. Fifth, interpreting the efficacy of phage therapy is usually complicated by simultaneous antibiotic therapy. While the success of phage therapy among patients with antibiotic-resistant infections is compelling, the majority of patients suitable for compassionate phage therapy in fact suffer from antibiotic sensitive chronic infections. Delineating the relative effects of antibiotic and phage therapy will be challenging, more so when the effect of these antimicrobials is not mutually exclusive and may often be synergistic [59]. The varying degrees of antibiotic susceptibility between patient’s bacterial pathogens in a potential trial cohort adds further complication. Lastly, alongside the usual disincentives for the development of antibiotics, the use of naturally occurring phage for therapy has aspects that disincentivise private investment. For example, naturally occurring phage are discoveries, not protectable, and easy to ‘copy’. This presents a financial challenge in taking phage therapy through the costly clinical trials system which is generally set up to meet the needs of sizeable businesses. Moreover, the provision of personalised treatments and phage for uncommon bacteria or niche clinical settings is unlikely to be financially attractive [60]. 

Recommendations for successful phage therapy clinical trials have been published elsewhere [61,62]. This analysis of existing trial data suggests that successful trials must carefully consider how to consistently deliver a therapeutic amount of the right phage(s) to the right place to treat infections containing enough susceptible bacterial cells. The results of any future trials which fail to demonstrate efficacy because of shortcomings in one or more of these areas will be detrimental and further add to the perplexity of those looking at the field and wondering where the discrepancy between observational and trial results arises. Success will require close collaboration between clinical and phage specialists. Clinical trials will be important in the development of certain phage therapeutics, particularly prophylactic commercial phage products—such as phage-coated medical devices. However, given the challenges that therapeutic phage presents to the existing clinical trial model, observational clinical reports must be given their deserved significance, especially when antibiotic resistant infections have been treated, when appraising the overall safety and efficacy of clinical phage therapy. 

## 5. Conclusions

Clinical and safety trials consistently demonstrate that the use of naturally occurring phage for therapy, by various routes of administration, is safe. Clinical trials also show that phage is efficacious when the right amount of the right phage(s) are delivered to the right place to treat infections containing enough susceptible bacterial cells. However, the alignment of this constellation of factors has proved tricky for previous clinical trials and, despite this and other challenges, it is hoped that future trials will deliver the compelling results much anticipated by the field. In the meantime, the evidence around the safety and efficacy of phage therapy is considered sufficiently reassuring for ongoing compassionate use when antibiotics are unable to meet clinical needs.

## Figures and Tables

**Table 1:** A summary of clinical and safety trials of phage therapy. Yes: ✓; no: ✗; unclear: ?. * The phage titre used was 6 × 10^5^ PFU/mL. Only one paper commented that there were likely insufficient bacteria to enable phage replication.

	**Trial**	**Were the Phages Administered**					**Efficacy?**
		**The right phage(s)?** Was in vitro efficacy demonstrated before treatment?	**To the site of infection?** Did the phages come into contact with the target bacteria? Could some bacteria have been inaccessible to phages?	**At the right time during the infection?** I.e., could the infection have resolved before phage therapy could work or was the contact time between bacteria and phage sufficient for phage replication?	**In sufficient quantity?** Was a significant number of phages (>10^6^ PFU/mL) administered and/or available at the infection site?	**To sufficient bacteria?** Was the bacterial population sufficient to enable substantial phage replication?	
**Historical trials** **(pre-2000)**	Bryant et al. (1965), [38]	✗	✗	✓	?	-	✗
	Wittig et al. (1966), [36]	✗	✗	✓	✓	-	✗
	Marcuk et al. (1971), [37]	?	✓	✗	?	-	✗
**Modern trials** **(post-2000)**	Rhoads et al. (2009), [21]	✗	✓	✓	✓	-	✗
	Wright et al. (2009), [19]	✓	✓	✓	✓*	-	✓
	Rose et al. (2014), [24]	✗	✓	✗	✓	-	✗
	Sarker et al. (2016), [25]	✗	✓	✓	✓	✗	✗
	Jault et al. (2019), [28]	✗	✓	✓	✗	-	✗
	Ooi et al. (2019), [29]	✓	✓	✓	✓	-	✓
	Leitner et al. (2021), [30]	✓	?	✓	✗	-	✗

## Data Availability

Data sharing is not applicable to this article as no datasets were generated or analysed during the current study.

## Supplementary Materials

The following supporting information can be downloaded at: https://www.mdpi.com/article/10.3390/antibiotics11101340/s1, File S1: PRISMA abstract checklist; File S2: PRISMA checklist; File S3: Trial data; File S4: Critical appraisal.

## Abbreviations

PRISMA: Preferred Reporting Items for Systematic Reviews and Meta-Analyses.
