# Supplementary material for: The Safety and Efficacy of Phage Therapy: A Systematic Review of Clinical and Safety Trials
Source: Antibiotics (Basel). 2022 Sep 30;11(10):1340. doi: 10.3390/antibiotics11101340 (PMC9598614; doi:10.3390/antibiotics11101340)
Supplement: Supplementary file 1 [file antibiotics-11-01340-s001.zip › Supplementary file S1.pdf]

Supplementary file S1: PRISMA 2020 for Abstracts Checklist

Section and Topic	Item #	Checklist item	Reported (Yes/No)
**TITLE**			
Title	1	Identify the report as a systematic review.	Y
**BACKGROUND**			
Objectives	2	Provide an explicit statement of the main objective(s) or question(s) the review addresses.	Y
**METHODS**			
Eligibility criteria	3	Specify the inclusion and exclusion criteria for the review.	N<br>Insufficient space in word limit of 200.
Information sources	4	Specify the information sources (e.g. databases, registers) used to identify studies and the date when each was last searched.	N<br>Insufficient space in word limit of 200.
Risk of bias	5	Specify the methods used to assess risk of bias in the included studies.	N<br>Insufficient space in word limit of 200.
Synthesis of results	6	Specify the methods used to present and synthesise results.	N/A
**RESULTS**			
Included studies	7	Give the total number of included studies and participants and summarise relevant characteristics of studies.	Y
Synthesis of results	8	Present results for main outcomes, preferably indicating the number of included studies and participants for each. If meta-analysis was done, report the summary estimate and confidence/credible interval. If comparing groups, indicate the direction of the effect (i.e. which group is favoured).	Y
**DISCUSSION**			
Limitations of evidence	9	Provide a brief summary of the limitations of the evidence included in the review (e.g. study risk of bias, inconsistency and imprecision).	N<br>Insufficient space in

Section and Topic	Item #	Checklist item	Reported (Yes/No)
			word limit of 200.
Interpretation	10	Provide a general interpretation of the results and important implications.	Y
**OTHER**			
Funding	11	Specify the primary source of funding for the review.	N/A
Registration	12	Provide the register name and registration number.	N/A

From: Page MJ, McKenzie JE, Bossuyt PM, Boutron I, Hoffmann TC, Mulrow CD, et al. The PRISMA 2020 statement: an updated guideline for reporting systematic reviews. BMJ 2021;372:n71. doi: 10.1136/bmj.n71
For more information, visit: http://www.prisma-statement.org/
