# Supplementary material for: The Safety and Efficacy of Phage Therapy: A Systematic Review of Clinical and Safety Trials
Source: Antibiotics (Basel). 2022 Sep 30;11(10):1340. doi: 10.3390/antibiotics11101340 (PMC9598614; doi:10.3390/antibiotics11101340)
Supplement: Supplementary file 1 [file antibiotics-11-01340-s001.zip › Supplementary file S3.pdf]

Supplementary file S3: clinical and safety trials of phage therapy

Report details		Clinical details					
**Author (year), [citation], location, study type**	**Participants and pathogen(s)**	**Condition details**	**Bacterial sensitivity to phage(s)**	**Phage treatment**	**Treatment schedule(s) and monitoring assays**	**Efficacy**	**Safety & adverse effects**
Bryant et al. (1965), [38]<br><br>United States<br><br>Single-centre, randomised, double-blinded, placebo-controlled trial	Placebo, n = 24<br>Phage, n = 24*<br><br>*22 children later received phage in a six-month observational study without a comparator; these data were excluded.<br><br>*S. aureus*	Recurrent furunculosis in schoolchildren.	No phage sensitivity testing reported.	Staphylococcal phage lysate (Gratia phage strain).	Phage or placebo (uninoculated culture broth) were administered by IM injection into the deltoid at weekly intervals for six months. An initial dose of 0.05ml was gradually increased until a dose of 0.5ml in week 5, which was maintained until the end of the trial. One patient in each group received antibiotics for orbital cellulitis.<br><br>Safety and efficacy was monitored by daily, three times weekly and once weekly clinical inspections by the housemother, nursing supervisor and study physician respectively. The number, size and location of boils was recorded. To investigate the immune response to *S. aureus*, serum anti-alpha haemolysin was investigated for 11 patients.	No significant decline in furunculosis relative to control.	'Reactions to the vaccine were limited to local erythema, pain and swelling' Initially one third of patients had 4cm erythema around the injection site. Later, these reactions decreased in severity and frequency.'
Wittig et al. (1966), [36]<br><br>United States<br><br>Single-centre, randomised, double-blinded, placebo-controlled trial	Placebo, n = 22<br>Phage, n = 22<br><br>N/A	Infective childhood asthma.	No phage sensitivity testing reported.	Staphylococcal phage lysate containing '10 billion active phage particles' per ml.	Phage or placebo (uninoculated culture broth) were administered by injection at weekly intervals for five months. An initial dose of 0.1ml was gradually increased until a dose of 0.5ml, which was maintained until the end of the trial. Patients continued to receive other therapeutic interventions including bronchodilators, mucolytics and antibiotics.<br><br>The parents of the patients were given symptom cards and recorded episodes of coughing, fever, wheezing and colds. Note, the five months of the study period covered winter 1963-64.	No significant difference in symptom days relative to control. Nasopharyngeal cultures taken at the end of the study did not reveal any difference between the prevalence of Staphylococcal cultures.	No comment.
Marcuk et al. (1971),	Placebo, n = 30<br>Phage, n = 30	Acute cholera.	Phage sensitivity reported,	Phage cocktails against the classical (4	Different treatment regimens were compared. First, 20ml (ages 7-12) to 25ml (ages 13+) of oral phage or placebo (Martin's peptone media),	Phage therapy did not offer significant improvement over	No comment.

[37]<br><br>Pakistan/USSR<br><br>Single-centre, randomised, placebo-controlled trial	*Vibrio cholerae*		unclear if pro- or retrospective.	phages) and El Tor (5 phages) strains of *V. cholerae*. Titre was 10^8-10^9 PFU/ml. Cocktails prepared by USSR Ministry of Health labs.	followed by 20-25ml of 2% bicarbonate, was administered alternately to each of 30 patients, such that 15 received phage and 15 placebo. The first dose was given 8-12h after admission, with two further doses during 24-48h.<br><br>An alternative phage treatment strategy was also tested and compared to tetracycline. Phage or tetracycline were given within 4h of admission. Fifteen patients received oral phage as before, with the addition of 15ml (ages 7-12) to 20ml (ages 13+) of IM phage injected at the initiation of oral phage therapy. The control cohort of 15 patients was treated with oral tetracycline.<br><br>Stool samples for quantitative analysis of Vibrios and phage were collected before treatment and at 4h, 12h and 24h thereafter and then once daily for three days. Stool output was measured every 4h, and urine every 8h, until the cessation of diarrhoea. Blood was drawn at the same time for haematocrit, specific gravity and plasma protein values. Rectal swabs for Vibrio were taken on admission and for 7 days thereafter. Pulse, blood pressure, respiratory rate and temperature were measured at 8h intervals.	standard rehydration therapy. Tetracycline was superior phage therapy.	
Bruttin & Brussow (2005), [20]<br><br>Switzerland<br><br>Single-centre, randomised, double-blinded, placebo-controlled, crossover safety trial	n = 15<br><br>N/A	Healthy adults.	N/A	*E. coli* phage T4 (Geneva University, Switzerland) in 150ml of mineral water (258mg/L HCO_3^-).<br><br>There were three treatments:<br>A) 10^5 PFU/ml<br>B) 10^3 PFU/ml;<br>C) placebo.	The study ran for 4 weeks. Week 1 captured two random baseline stool samples. During weeks 2-4 the subjects randomly received one of the three oral treatments 3 times per day for 2 days, followed by 5 days washout with mineral water. A randomised crossover design was used for treatment allocation.<br><br>All stools produced during the three test weeks were analysed for the presence of T4 phage. Serum samples taken before and after the study were used to investigate: 1) the presence of anti-T4 IgG, IgA or IgM by ELISA; 2) liver function tests (AST and	N/A<br>Safety trial	All treatments were well tolerated. Five mild adverse events were reported. There was no association of adverse events with either phage treatment or placebo. These were rated by the study physician as unrelated to the study interventions. All liver function tests remained normal. No anti-T4 antibody responses were detected at the end of the

Reg: N/A					ALT); 3) the presence of phage in serum. Participants were clinically examined at the start and end of the study period. Patient self-reporting of adverse events was collated using forms.		study period.
Rhoads et al. (2009), [21]<br><br>United States<br><br>Phase I randomised, double-blinded, placebo-controlled, safety trial<br><br>Reg: NCT00663091	Phage, n = 18<br>Placebo, n = 21<br><br>N/A	Chronic venous leg ulcer patients with or without infection.	N/A	Phage cocktail WPP-201.<br><br>Contains eight types of phage active against *S. aureus, P. aeruginosa* and *E. coli*, each at 10^9 PFU/ml and suspended in phosphate-buffered saline.	Test group: 4ml of WPP-201 in 46ml of saline.<br>Control group: sterile saline only<br>Administered topically once weekly for 12 weeks by ultrasonic debridement machine at a drip rate of 200ml/h.<br><br>Dressings (Promogran, Acticoat, Allevyn and three-layer compression bandages) were applied with Bovine lactoferrin (1%) and xylitol (5%) topical gel.<br><br>Antibiotic administration was permitted where signs of acute inflammation were observed.<br><br>Efficacy was assessed by weekly assessments of wound size and healing as well as photographs. Bacterial wound cultures were examined bi-weekly. Outcome was assessed at 12 weeks based on degree of closure with follow up at 16 and 24 weeks.<br><br>Safety was monitored weekly by vital signs and patient self-reporting and bi-weekly by blood tests including 'blood chemistries and blood cell counts'.	No significant difference in the rate or frequency of wound healing was observed between the control and test groups.<br><br>The paper did not evaluate changes in bacterial cultures.<br><br>The trial was not primarily designed as an efficacy study. Signs or microbiological evidence of infection were not inclusion criteria.	'No adverse effects were attributed to the study product'. No significant differences in the quantity or quality of adverse effects was observed between the two groups.<br><br>'Our observation that WPP-201 was safe and caused no adverse events was not unexpected given the ubiquity of bacteriophages in most environments, including the human skin, mouth and gastrointestinal tract, and the long history of their safe use in many clinical disciplines, including the treatment of infected wounds'
Wright et al. (2009), [19]<br><br>UK<br><br>Phase I/II randomised, double-blinded, placebo-controlled, safety trial	Phage, n = 12<br>Placebo, n = 12<br><br>'Antibiotic resistant' *P. aeruginosa*	Chronic otitis.	Sensitivity confirmed.	A cocktail of 6 anti-Pseudomonal phages (NCIMB deposit numbers 41174-41179, UK), each at 10^4 PFU/ml, suspended in 10% glycerol in phosphate buffered saline.	Test group: one intra-aural dose of 0.2ml of phage cocktail<br>Control group: one intra-aural dose of 0.2ml of glycerol/PBS diluent<br><br>No other treatments, including antibiotics, were used.<br><br>Patients were followed up at days 7, 21 and 42 after treatment.	Significant clinical improvements from baseline were observed in the test group only. Bacterial counts were significantly lower in the test group only.	'No treatment-related adverse effects were reported' as determined by the study clinician.<br><br>'There were no reportable side effects and no evidence of local or systemic toxicity'.

Reg: 2004-001691-39					Outcomes were variously assessed using patient daily diary cards, monitoring of temperature before administration and 6h after treatment and at follow up visits by patient self-reporting of adverse events, patient and clinician completed visual analogue scales, digital otoscopy, audiogram, bacteria and phage counts.		
Sarker et al. (2012), [22]<br><br>Bangladesh<br><br>Single-centre, randomised, double-blinded, placebo-controlled, crossover safety trial<br><br>Reg: N/A	n = 15<br><br>N/A	Healthy adults.	N/A	A cocktail of 9 T4-like E. coli phages (Nestle phage collection) in 150ml of mineral water (258mg/L HCO_3^-).<br><br>There were three treatments:<br>A) 3 x 10^7 PFU<br>B) 3 x 10^9 PFU;<br>C) placebo.	The study ran for 4 weeks. Week 1: baseline physical examination and stool and blood samples. Each week thereafter subjects randomly received 50ml of one of the three oral treatments 3 times per day for 2 days, followed by 5 days washout with mineral water. A randomised crossover design was used for treatment allocation.<br><br>Stool samples and vital signs were taken during phage treatment. On the first day after phage treatment blood and stool samples were taken and a physical examination was performed. Blood tests were: biochemistry, LFTs, U&Es, FBC, CRP, platelet, haematocrit. The presence of LPS or phage specific antibodies (IgA, IgM, IgG) in serum was investigated. Stool samples were used to investigate phage titres. Patients were questioned regarding gastrointestinal complaints.	N/A<br>Safety trial	'No adverse events were observed by self-report, clinical examination, or from laboratory tests for liver, kidney, and hematology function.'

McCallin *et al.* (2013), [23]<br><br>Bangladesh<br><br>Single-centre, randomised, double-blinded, placebo-controlled, crossover safety trial<br><br>Reg: N/A	n = 15<br><br>N/A	Healthy adults (n = 5)<br>Healthy children:<br>Aged 5-10, (n = 5)<br>Aged <5, (n = 5)	N/A	A commercially available 17-phage cocktail (ColiProteus; Microgen, Russia) in 150ml (adults) or 75ml (children) of mineral water (258mg/L HCO_3^-).<br><br>There were three treatments:<br>A) 7 x 10^6 PFU/ml<br>B) 7 x 10^5 PFU/ml;<br>C) placebo.	The study was conducted as described in Sarker et al. 2021. Baseline physical examination and stool and blood samples were undertaken. Each week thereafter subjects randomly received one of the three oral treatments 3 times per day for 2 days, followed by 5 days washout with mineral water. A randomised crossover design was used for treatment allocation.<br><br>Stool samples and vital signs were taken during phage treatment. On the first day after each phage treatment blood and stool samples were taken and a weekly physical examination was performed. Blood tests were: biochemistry, LFTs, U&Es, FBC, CRP, platelet, haematocrit. The presence of phage specific antibodies (IgA, IgM, IgG) in serum was investigated. Stool samples were used to investigate phage titres.	N/A<br>Safety trial	This study 'did not associate adverse effects with oral phage exposure'.

Supplementary file S3: clinical and safety trials of phage therapy

Rose et al. (2014), [24]<br><br>Belgium<br><br>Investigator-driven clinical trial<br><br>Reg: N/A	n = 9<br><br>Multi-drug resistant *P. aeruginosa* and/or *S. aureus* infection.	Burn-wound infection. One patient had two burn wound infections.	No phage sensitivity testing reported.	'BFC-1' cocktail, containing lytic phages active against *P. aeruginosa* (14/1 and PNM) and *S. aureus* (ISP), each at 10^9 PFU/ml.	BFC-1 was applied in a single topical dose at approximately 1ml per $50cm^2$.<br><br>The cocktail was applied to half of a patient's wound area, the other half of the wound received standard care. Punch biopsies of each half of the wounds were taken to investigate bacterial load before and 2-4h after BFC-1 therapy. Five phage applications took place during surgery, prior to which wounds had been cleansed with 5% Hibitane and filtered water. Average area of phage administration was $95cm^2$ (range, $25\text{-}150cm^2$).<br><br>Patients with *P. aeruginosa* were also treated with one dose (25mg/kg initially) of amikacin in combination with ceftazidime (1g initially) or meropenem (2g/8h). Patients with *S. aureus* infection were also treated with vancomycin (1g initially) or linezolid (1200mg/day).<br><br>Adverse event monitoring: 'patient medical files were screened for adverse events, clinical abnormalities and changes in laboratory results. Clinical abnormalities that were screened for included cardiovascular, renal and respiratory complications and pain. 'Clinical laboratory tests included the blood formula and standard haemostasis, biochemical, pharmacological and toxicological parameters'.	The bacterial loads were found to be very low in 8/10 wounds, owing to an up to 7-day delay in admission to the trial during which patients received intensive antibiotic therapy.<br><br>In all cases, the biopsy bacterial load was unchanged after phage application.<br><br>The authors note that they did 'not [expect] that a one-off application [...] on a small wound surface would generate conclusive proof of the efficacy'. Furthermore, this 'trial did not allow for an adequate evaluation of the efficacy of the phage cocktail'.	'No adverse effects, clinical abnormalities or changes in laboratory results that could be related to the application of phages were observed.'
Sarker *et al.* (2016), [25]<br><br>Bangladesh<br><br>Single-centre, randomised, double-blinded, placebo-controlled safety trial	Phage (T4), n = 38<br>Phage (Microgen), n = 40<br>Placebo, n = 37<br><br>About half the patients were found had stool	6- to 24-month-old male children with acute bacterial diarrhoea of <48h duration	Retrospective analysis only.	The treatments were:<br><br>1.4 x 10^9 PFU of commercially available >17-phage cocktail (ColiProteus; Microgen, Russia).	Patients who had received or needed antibiotic treatment were excluded.<br><br>Oral phage in addition to standard treatment or just standard treatment (placebo; reduced osmolarity oral rehydration solution supplemented with zinc) was given 3 times daily for 4 days.<br><br>The frequency, consistency and volume of stool and urine were recorded. Daily stool samples were	Intestinal amplification of phages was not observed, including in patients with susceptible *E. coli*. The authors considered there may have been too little *E. coli* to sustain phage replication. The phage coverage of bacterial	'No adverse events attributable to oral phage application were observed (primary safety outcome)'.

Reg: NCT00937274	samples positive for *E. coli*. Antibiotic sensitivities not performed.			3.6 x 10^8 PFU of a cocktail of 11 T4-like *E. coli* phages (Nestle phage collection, Switzerland).	obtained for culture and phage titre. Safety analyses were: clinical examination and patient self-reporting. Blood tests: biochemistry, LFTs, U&Es, FBC, CRP, platelet, haematocrit.	strains may have been insufficient. Moreover, some patients were found to have Streptococcal overgrowth instead of *E. coli*. Finally, no stomach acid neutralisation (HCO_3^-) was used. Phages are often very susceptible to stomach acid.	
Sarker *et al.* (2017), [26]<br><br>Bangladesh<br><br>Single-centre, randomised, placebo-controlled, crossover phase I safety trial<br><br>Reg: N/A	n = 40<br><br>N/A	Healthy children: Aged 5-9, (n = 20) Aged 9m to 5y, (n = 20)	N/A	The treatments were:<br><br>A cocktail of 9 T4-like *E. coli* phages (Nestle phage collection, Switzerland) was used at two doses (10^6 or 10^8 PFU) in children aged 5-9 and at two reduced doses in children aged 9m to 5y (10^5 or 10^7 PFU).<br><br>A commercially available 17-phage cocktail (ColiProteus; Microgen, Russia) used at two doses (0.5 or 1 x 10^9 PFU) in children with a median age of 5y..	The study ran for 3 weeks. Three days beforehand baseline physical examination and stool and blood samples were taken. For each of three weeks thereafter 15 children from each age category randomly received either placebo or a low or high dose of the oral T4-like phage cocktail for 3 times per day for 2 days, followed by 5 days washout. Additionally, each week the remaining 5 children from each age category randomly received either placebo or a low or high dose of the oral ColiProteus phage cocktail for 3 times per day for 2 days, followed by 5 days washout.<br><br>Stool samples were obtained for phage titre and/or culture during phage treatment and after washout. Blood samples were taken after phage treatment to investigate serum LPS, serum phage and the blood panels: clinical biochemistry, LFTs, U&Es, FBC, CRP, platelet, haematocrit. The presence of LPS or phage specific antibodies (IgA, IgM, IgG) in serum was investigated using serum taken at the end of the 3 week period.	N/A<br>Safety trial	'No adverse effects of phage application were seen clinically and by clinical chemistry'.

Supplementary file S3: clinical and safety trials of phage therapy

McCallin *et al.* (2018), [27]<br><br>Bangladesh<br><br>Single-centre, randomised, placebo-controlled, crossover phase I safety trial<br><br>Reg: N/A	n = 21<br><br>Positive for *S. aureus* colonisation.	Healthy adults.	N/A	The treatments were:<br><br>Staphylococcal monophage preparation at 10^6 PFU/ml (Eliava, Georgia).<br><br>Pyophage cocktail at 10^5 PFU/ml (Eliava, Georgia).	Ten subjects received either oral phage preparation or placebo at 10ml three times daily for two days, followed by a washout period. The remaining two treatments were then administered in a randomised order for each of the next two weeks. Eleven patients underwent the same process, but with the treatments administered nasally. A randomised crossover design was used for treatment allocation.<br><br>Blood samples, temperature and vital signs were taken at baseline and after each of the three treatments. The blood panels comprising 30 individual assays were: clinical biochemistry, LFTs, U&Es, FBC, CRP, platelet, haematocrit. Patients and their relatives were encouraged to self-report any adverse effects.	N/A<br>Safety trial	'The lack of [treatment-related] adverse effects in any treatment groups supports the clinical safety of *S. aureus* phages administered as a single phage or as phage cocktail'.
Jault *et al.* (2019), [28]<br><br>France & Belgium<br><br>Multi-centre, randomised, controlled, double-blind phase I/II trial<br><br>Reg: NCT02116010	Phage, n = 10<br>Placebo, n = 12<br><br>*P. aeruginosa*<br>Varying antibiotic susceptibility.	Burn-wound infection.	Retrospective analysis only.	PP1131 cocktail (12 lytic phages; Pherecydes Pharma, France)<br><br>Concentration planned as 10^6 PFU/ml but deteriorated to 10^2 PFU/ml (considered subtherapeutic).	Once daily topical application of either PP1131 or standard of care 1% sulfadiazine silver emulsion cream for 7 days. Adjunctive antibiotic therapy at the discretion of the treating physician according to French Burn society recommendations. Patients were followed up for 14 days thereafter.<br><br>Safety assessments: clinical laboratory tests (haematology, chemical, and urine analyses), vital signs, physical examinations and clinical assessment of burn wounds.<br><br>Adjunctive antibiotics were permitted during the treatment period at the discretion of the treating physician.	Primary outcome was time taken for a sustained reduction in bacterial burden of two quadrants or more assessed by semi-quantitative culture results:<br><br>Phage group: median 144h (95% CI, 48h to not reached)<br><br>Control group: 47h (95% CI, 23-122h)<br><br>Amongst those in the phage group who did not reach the primary endpoint 3/10 had resistant colonies.	'Adverse events' reported among 23% of phage patients and 54% of control patients. No substantial difference between the groups.<br><br>Although the phage titre was 10^2 PFU/ml, the equivalent of 10^6 phage particles (most inactive) were applied to patients and 'did not provoke safety issues'.

Supplementary file S3: clinical and safety trials of phage therapy

Ooi *et al.* (2019), [29]<br><br>Australia<br><br>Phase I open-label, single-centre, multiple ascending dose trial<br><br>Reg: ACTRN12616000002482	n = 9<br><br>*S. aureus*	Chronic rhinosinusitis.	Sensitivity confirmed.	Cocktail of 3 *S. aureus* phages (AB-SA01; AmpliPhi, Australia).	The 9 patients were divided into cohorts of 3. Cohort 1 received twice daily intranasal irrigations of 3 x 10^8 PFU for 7 days. Cohort 2 received twice daily intranasal irrigations of 3 x 10^8 PFU for 14 days. Cohort 3 received twice daily intranasal irrigations of 3 x 10^9 PFU for 14 days. Patients taking oral antibiotics, corticosteroids or who had used oral antibiotics within the last month were not eligible for the trial.<br><br>Safety and efficacy were monitored using clinical assessments: Sino-Nasal Outcome Test–22; Visual Analogue Scale; Lund-Kennedy Score derived from trial entry and exit endoscopic assessment. Vital signs were observed before treatment and 0.5 and 2.0 hours post treatment. Patients were asked to monitor their own temperature twice daily. Pre- and post-treatment bacterial cultures were assessed. Blood samples for FBC, haematocrit, clinical biochemistry, U&Es and LFTs were taken pre- and post- trial. Patients were followed up 7 days after the trial for additional adverse effect reporting.	The primary outcome was safety and tolerability.<br><br>All patients had a reduction in *S. aureus* growth, and 2 of 9 had clinical and microbiological evidence of eradication of infection.	'The AB-SA01 phage cocktail for intranasal irrigation appears to be safe and well tolerated to 3 × 10^9 PFU for 14 days with no dose-limiting adverse effects.'
Gindin *et al.* (2019), [17]<br><br>Febvre *et al.* (2019), [18]<br><br>United States<br><br>Single-centre, randomised, double-blinded, placebo-controlled crossover trial<br><br>Reg: NCT03269617	n = 32<br><br>N/A	Healthy adults with 'mild to moderate gastrointestinal distress but no diagnosable gastrointestinal conditions'.	No phage sensitivity testing reported.	Cocktail of 4 *E. coli* phages (PreforPro; Deerland Enzymes, United States)	The study ran for 6 weeks. The treatment was a capsule containing 10ng of phages in an inert carrier. The control was a capsule containing only inert carrier. Participants were randomly assigned to receive a once-daily phage or control capsule for 4 weeks, followed by a 2 week washout and then 4 weeks of the opposite capsule. A randomised crossover design was used for treatment allocation.<br><br>Efficacy was investigated by patient self-reporting at the start and end of each treatment period. Safety was monitored using blood and stool samples taken at the start and end of each 4-week treatment period. Blood tests among the 32 patients were: LFTs, U&Es, calcium, glucose, total CO_2. Additional blood tests were subsequently	Gindin *et al*: the primary outcomes were the safety and tolerability of phage.<br><br>'Participants also reported significant improvements on several symptoms of gastrointestinal distress while taking both the treatment and placebo'.	Gindin *et al*: There 'were no reports of adverse events during the trial'. Phage was 'both safe and tolerable'. 'All mean values remained within clinically acceptable ranges.' 'All [metabolic] measurements remained within clinically acceptable ranges after 28 days of consumption, highlighting its safety in a human population' and showing that the phages were 'safe for daily human consumption'.

					reported for 36 participants, reflecting inclusion of an additional 4 who only completed one study arm: plasma lipids, CRP, GM-CSF, IFNγ, IL-1α, IL-2, IL-4, IL-5, IL-6, IL-7, IL-8, IL-10, IL-12, IL-13 and TNFα. Stool assays were reported for the cohort of 36 patients: calprotectin, secretory IgA, trigylcerides, short-chain fatty acids and molecular analysis of the microbiome.		Febvre *et al*: there were no significant stool assay, cytokine or CRP changes except that phage consumption was associated with a small but significant decrease in IL-4.
Leitner *et al.* (2021), [30]<br><br>Georgia<br><br>Randomised, placebo-controlled, double-blind trial<br><br>Reg: NCT03140085	Placebo, n = 32<br>Phage (Pyophage), n = 28<br>Antibiotics, n = 37<br><br>Pathogens were *Enterococcus* spp., *E. coli*, *Proteus mirabilis*, *P. aeruginosa*, *Staphylococcus* spp. or *Streptococcus* spp.	Men scheduled for transurethral resection of the prostate (TURP), with complicated UTI or recurrent uncomplicated UTI but no signs of systemic infection.	Sensitivity confirmed.	Pyophage cocktail (Eliava Institute, Tbilisi). Contains phages against *Streptococcus* spp., *Staphylococcus* spp., *Proteus* spp., *Escherichia coli*, *P. aeruginosa* and *Enterococcus* spp.<br><br>All phages were present in Pyophage at >10^5 PFU/ml, except phages against *Streptococcus* spp. (>10^4 PFU/ml).	Patients received twice daily intra-vesicular administration of 20ml of Pyophage or placebo or systemic antibiotics for 7 days immediately after TURP. The solution was kept in the bladder for 30-60 minutes each time. No antibiotic prophylaxis was given to either the Pyophage or placebo group. The group receiving antibiotics did so according to appropriate antibiotic sensitivities. Before TURP and after the 7-day treatment period, urinalysis, urine culture and an International Prostate Symptom Score were performed.<br><br>Safety monitoring included clinical examination and patient self-reporting, including a bladder and pain diary.	Rates of treatment success did not differ between the three groups.	'No significant difference in the type or severity of adverse events, including fever, was observed between any of the treatment groups. This is in line with all reports of bacteriophage therapy to date'
