# Supplementary material for: The Safety and Efficacy of Phage Therapy: A Systematic Review of Clinical and Safety Trials
Source: Antibiotics (Basel). 2022 Sep 30;11(10):1340. doi: 10.3390/antibiotics11101340 (PMC9598614; doi:10.3390/antibiotics11101340)
Supplement: Supplementary file 1 [file antibiotics-11-01340-s001.zip › Supplementary file S4.pdf]

Supplementary file S4: critical appraisal

Critical appraisal of manuscripts was performed using the Joanna Briggs Institute suite of critical appraisal tools available from https://joannabriggs.org/critical-appraisal-tools [accessed 22/12/2021].

Clinical trials

Author (year)	True randomisation?	Allocation to treatment groups concealed?	Treatment groups similar at baseline?	Participants blind to assignment?	Treatment givers blind to assignment?	Outcome assessors blind to assignment?	Treatment groups treated identically - other than the intervention	Complete follow up?	Participants analysed in allocated groups?	Consistent outcome measurement?	Reliable outcome measurement?	Appropriate statistical analysis?	Was the trial design appropriate?	Comment
Bryant et al. (1965), [38]<br><br>United States<br><br>Single-centre, randomised, double-blinded, placebo-controlled trial	Y	Y	Y	Y	Y	Y	Y	Y	Y	Y	N*	Y	Y	*Changes in boil frequency, character and size could reflect the activity of other pathogens.
Wittig et al. (1966), [36]<br><br>United States<br><br>Single-centre, randomised, double-blinded, placebo-controlled trial	U	U	Y	U	U	Y	Y	Y	Y	Y	N*	Y	Y	*The trial was conducted in winter. Symptom cards were used for recording. The respiratory symptoms recorded in either group could have been caused by a wide range of pathogens.

Marcuk et al. (1971), [37]<br><br>Pakistan/USSR	N*	U	Y	U	U	U	Y	Y	Y	Y	Y	Y	Y	*Patients allocated to phage or placebo groups on an alternate basis.
Bruttin & Brussow (2005), [20]<br><br>Switzerland<br><br>Single-centre, randomised, double-blinded, placebo-controlled safety trial<br><br>Reg: N/A	U	Y	Y	U	U	Y	Y	Y	Y	Y	Y	Y	Y	-
Rhoads et al. (2009), [21]<br><br>United States<br><br>Phase I randomised, double-blinded, placebo-controlled, safety trial<br><br>Reg: NCT00663091	Y	Y	Y	U	U	U	Y	Y	Y	Y	Y	Y	Y	-
Wright et al. (2009), [19]<br><br>UK<br><br>Phase I/II randomised, double-blinded, placebo-controlled, safety trial<br><br>Reg: 2004-001691-39	Y	Y	Y	Y	Y	Y	Y	Y	Y	Y	Y	Y	Y	-
Sarker et al. (2012), [22]<br><br>Bangladesh	U	U	Y	U	U	U	Y	Y	Y	Y	Y	Y	Y	-

Single-centre, randomised, double-blinded, placebo-controlled safety trial<br><br>Reg: N/A														
McCallin *et al.* (2013), [23]<br><br>Bangladesh<br><br>Single-centre, randomised, double-blinded, placebo-controlled safety trial<br><br>Reg: N/A	U	Y	Y	U	U	U	Y	Y	Y	Y	Y	Y	Y	-
Rose et al. (2014), [24]<br><br>Belgium<br><br>Investigator-driven clinical trial<br><br>Reg: N/A	N/A	N/A	Y	U	U	U	Y	Y	Y	Y	Y	Y	Y	-
Sarker *et al.* (2016), [25]<br><br>Bangladesh<br><br>Single-centre, randomised, double-blinded, placebo-controlled safety trial<br><br>Reg: NCT00937274	Y	Y	Y	Y	Y	Y	Y	Y	Y	Y	Y	Y	Y	-
Sarker *et al.* (2017), [26]<br><br>Bangladesh	U	Y	Y	U	U	U	Y	Y	Y	Y	Y	Y	Y	-

Single-centre, randomised, placebo-controlled<br>phase I safety trial<br><br>Reg: N/A														
McCallin *et al.* (2018), [27]<br><br>Bangladesh<br><br>Single-centre, randomised, placebo-controlled<br>phase I safety trial<br><br>Reg: N/A	Y	Y	Y	Y	Y	U	Y	Y	Y	Y	Y	Y	Y	-
Jault *et al.* (2019), [28]<br><br>France & Belgium<br><br>Multi-centre, randomised, controlled, double-blind phase I/II trial<br><br>Reg: NCT02116010	Y	Y	Y	Y	N	Y	Y	Y	Y	Y	Y	Y	Y	-
Ooi *et al.* (2019), [29]<br><br>Australia<br><br>Phase I open-label, single-centre, multiple ascending dose trial<br><br>Reg: ACTRN12616000002482	N	U	Y	U	U	U*	Y	Y	Y	Y	Y	Y	Y	*Endoscopy videos were scored by a blinded surgeon
Gindin *et al.* (2019), [17]<br><br>United States	U	U	Y	Y	U	U	Y	Y	Y	Y	Y	Y	Y	-

Single-centre, randomised, double-blinded, placebo-controlled trial<br><br>Reg: NCT03269617														
Leitner *et al.* (2021), [30]<br><br>Georgia<br><br>Randomised, placebo-controlled, double-blind trial<br><br>Reg: NCT03140085	Y	Y	Y	Y*	Y*	Y	Y	Y	Y	Y	Y	Y	Y	*Phage and placebo were blinded, antibiotics were not blinded
